# Hyperopic shift caused by capsule contraction syndrome after microincision foldable intraocular Lens implantation: case series

**DOI:** 10.1186/s12886-019-1117-y

**Published:** 2019-05-20

**Authors:** Tae Gi Kim, Sang Woong Moon

**Affiliations:** Department of Ophthalmology, Kyung Hee University Hospital at Gangdong, Kyung Hee University, # 892, Dongnam-ro, Gangdong-gu, Seoul, 05278 Republic of Korea

**Keywords:** Capsule contraction syndrome, Microincision cataract surgery, Refractive change, Nd:YAG laser anterior capsulotomy, Intraocular lens

## Abstract

**Background:**

Increasing interest in microincision cataract surgery has led to the use of more flexible intraocular lens (IOL). Flexible IOL may cause more IOL deformation and refractive error when capsule contraction syndrome (CCS) occurred. In this retrospective observational case series study, the aim was to report four cases of hyperopic shift caused by CCS after phacoemulsification with microincision foldable intraocular lens implantation.

**Case presentation:**

All of four patients underwent phacoemulsification and in-the-bag implantation of an Akreos MI60 (Bausch and Lomb) IOL from 2010 to 2016 in our clinic. These patients had been diagnosed with CCS and had undergone Nd:YAG laser anterior capsulotomy. The mean age of the patients with CCS was 66.8 ± 6.7 years and the mean time for development of CCS after the cataract surgery was 9.3 ± 6.9 months. The mean spherical equivalent (SE) value at the time of the CCS diagnosis was 0.88 ± 0.91 D, which had shown a hyperopic shift compared to the SE value of − 0.91 ± 1.29 D after cataract surgery. The mean SE decreased by − 0.47 ± 1.14 D after Nd:YAG laser anterior capsulotomy. The mean age, axial length, anterior chamber depth, and preoperative SE were not significantly different between the patient with CCS and the patients without CCS.

**Conclusions:**

In the case of IOL implantation with flexible materials in microincision cataract surgery, CCS can cause a hyperopic shift. Refractive error caused by CCS can be effectively corrected by Nd:YAG laser anterior capsulotomy.

## Background

Capsule contraction syndrome (CCS) is a common postoperative complication of cataract surgery. CCS is caused by lens epithelial cell proliferation and fibrosis leading to capsule shrinkage and contraction of the capsulorhexis opening as the anterior lens capsule becomes thicker and turbid. CCS is known to occur frequently in patients with weak zonules, diabetes, retinitis pigmentosa, pseudoexfoliation syndrome, uveitis, and high myopia [1–4]. Surgical factors associated with CCS include a small size of the capsulorhexis and the presence of lens epithelial cells in the anterior capsule [5]. CCS cause instability of the capsular bag, which may damage the lens zonule and cause intraocular lens decentration and changes in refractive power [6]. Therefore, capsule phimosis can affect vision because of opacification of capsule, intraocular lens (IOL) dislocation such as tilting and deformation.1 Because of the increasing use of multifocal IOLs and toric IOLs these days, IOL decentration and tilting of IOL can be critical for IOL function.

The occurrence of CCS has been reported with the use of various IOL materials such as polymethylmethacrylate, silicone, and acrylic and there have been a few reports that the material and design of IOLs have a strong influence on the occurrence of CCS [3, 7–20]. However, mechanically, IOL deformation and refractive changes due to capsule shrinkage can be more frequent when using an IOL made of flexible material in microincision cataract surgery. To our knowledge, there are few studies on refractive error after CCS and refractive errors changed after CCS treatment procedure [7, 10].

In this study, we reviewed patients who underwent phacoemulsification with in-the-bag implantation of a single-piece hydrophilic IOL (Akreos MI60, Bausch and Lomb, Rochester, New York, USA) on demographics, refractive change according to CCS development, and reported 4 cases of hyperopic shift caused by CCS that were effectively treated with Nd:YAG laser anterior capsulotomy. In this study, CCS was defined as the exaggerated extreme reduction in diameter and fibrosis of anterior capsulorhexis opening following cataract removal. Anterior capsulorhexis opening with capsular phimosis was identified on slit-lamp examination.

## Case presentation

This retrospective clinical study includes 299 eyes (238 patients) who underwent cataract surgeries with in-the-bag implantation of an Akreos MI60 (Bausch and Lomb, Rochester, New York, USA) IOL, at our clinic between January 2010 and December 2016.

One surgeon (S.W.M) had performed the surgical procedure under 0.5% proparacaine hydrochloride topical anesthesia. The procedure was performed after creating a self-sealing 2.80 mm superior clear corneal incision adjacent to the limbus. The size of the continuous curvilinear capsulorhexis was approximately 5.5–6.0 mm in diameter. Phacoemulsification was performed with the phaco chop technique using the AMO Sovereign Compact-WhiteStar System (Advanced Medical. Optics, Santa Ana, CA) followed by in-the-bag implantation of a single-piece hydrophilic IOL (Akreos MI60).

Mann-Whitney U-tests were used to compare age, axial length, anterior chamber depth, and pre- and postoperative refractive spherical equivalent (SE) between patients with and without CCS. We also compared the refractive changes, UCVA, and BCVA of patients with CCS and those without CCS.

Of the 299 eyes, 4 eyes of 4 patients had been diagnosed with CCS. There was no statistically significant difference in demographics, ocular parameters, pre- and postoperative SE between patients with CCS (*n* = 4) and patients without CCS (*n* = 295)(Table 1). The demographics, refractive changes, UCVA, and BCVA of patients with CCS were summarized in Table 2. Mean SE was − 0.91 ± 1.29 D after cataract surgery and 0.88 ± 0.91 D after CCS occurrence which showed hyperopic shift compare with postoperative value and those who without CCS. After the Nd: YAG laser anterior capsulotomy, it was decreased to − 0.47 ± 1.14D.

**Table 1:** Comparison of clinical characteristics between the patients with and without capsular contraction syndrome (CCS)

	CCS (+) (*n* = 4)	CCS (−) (*n* = 295)	*p* value^*^
Age	66.8 ± 6.7	69.6 ± 9.35	0.469
Sex (M:F)	1:3	103:192	
AL (mm)	22.6 ± 1.7	23.0 ± 1.03	0.375
ACD (mm)	3.04 ± 0.53	3.08 ± 0.47	0.801
Pre-op SE (D)	−1.03 ± 1.44	−0.87 ± 1.95	0.318
Post-op 1Mo SE (D)	−0.91 ± 1.29	−0.62 ± 1.04	0.444
Post-op 12Mo SE (D)	+ 0.88 ± 0.91^a^	- 0.60 ± 1.13	0.038

*AL* Axial length, *ACD* anterior chamber depth, *SE* Spherical equivalent

^*^p value by Mann-Whitney tset

^a^SE after CCS

**Table 2:** Clinical characteristics of patients who developed capsular contraction syndrome (CCS) following cataract surgery

Case	Age	Sex	AL (mm)	ACD (mm)	Spherical equivalent (D)				Months to CCS diagnosis from cataract surgery	Systemic disease
					Pre-op	Post-op	After CCS	After YAG		
1	60	F	21	3.09	- 2.00	- 0.125	+ 1.375	+ 0.25	11	DM, HTN
2	65	M	24.7	3.75	- 2.50	- 2.00	+ 0.375	- 1.375	18	–
3	66	F	23.2	2.85	- 0.125	- 2.00	- 0.125	- 1.50	2	–
4	76	F	21.5	2.5	+ 0.50	+ 0.50	+ 1.875	+ 0.75	6	DM, HTN
Mean	66.8 ±6.7	–	22.6 ± 1.7	3.04 ± 0.53	−1.03 ± 1.44	−0.91 ± 1.29	+ 0.88 ± 0.91	−0.47 ± 1.14	9.3 ± 6.9	–

*AL* Axial length, *ACD* Anterior chamber depth, YAG: Nd:YAG laser anterior capsulotomy

### Case 1

A 60-year-old woman presented with decreased visual acuity in the right eye. She was on regular follow-up at our clinic due to non-proliferative diabetic retinopathy and hypertension. She had undergone microincision phacoemulsification and an in-the-bag implantation of an Akreos MI60 IOL at our clinic 11 months ago.

**Fig. 1:**
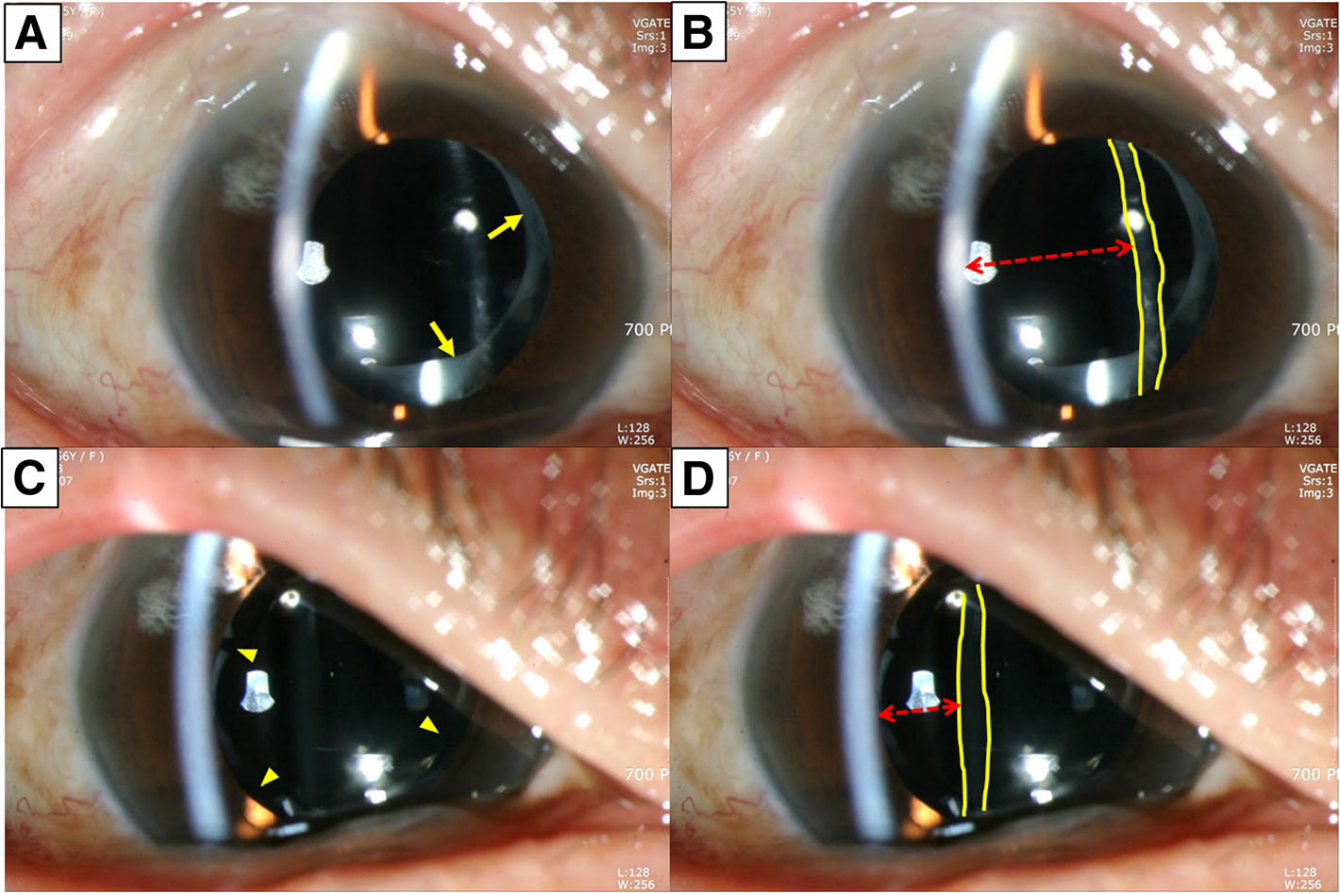
Anterior photographs of case 1 before (**a**, **b**) and after (**c**, **d**) Nd:YAG laser treatment. **a** Slit lamp examination shows contraction of the capsulorhexis opening and a thick anterior lens capsule margin (yellow arrow). **b** The intraocular lens (yellow line) reveals posterior bowing and an unusual deepening of the anterior chamber is observed (red arrow). **c** After relaxing the phimosis via the Nd:YAG laser anterior capsulotomy (yellow arrowhead), contraction of the capsulorhexis opening was released. **d**) The intraocular lens (yellow line) is flat and the anterior chamber depth (red arrow) is decreased compared to before the Nd:YAG laser treatment

At 1 month after the cataract surgery, her UCVA and BCVA was 20/20 with a SE of − 0.125 D. At the time of presentation, her UCVA was 20/32 and BCVA was 20/25, in the right eye. In the refraction test, the SE showed a hyperopic shift of + 1.375 D. Her intraocular pressure (IOP) was within the normal limit. A slit lamp examination after pupil dilation revealed anterior capsule contraction syndrome with a markedly thickened anterior capsule (Fig. 1). The IOL remained stable centrally in the capsular bag; however, it showed a slight posterior vaulting (Fig. 1). The fundus examination showed no definite change in the retina. The Nd:YAG laser anterior capsulotomy was performed by creating symmetrical incisions along four axes that radiated from the pupil center under local anesthesia in the right eye (laser energy = 1.5 mJ). The capsulotomy was created from the continuous curvilinear capsulorhexis margin to the IOL optical margin. Radial tearing should be considered when performing the initial incision. The incision was performed up to 0.5–1.0 mm from the IOL optical margin. Incisions over IOL haptics should be avoided because asymmetrical lens tilting can occur. One month after the Nd:YAG treatment, her UCVA and BCVA improved to 20/20, and the SE reduced to + 0.25 D. Six months later, her BCVA was 20/20 in the right eye, without any CCS.

### Case 2

A 65-year-old man complained of a progressive decrease in vision in his right eye. The patient had undergone phacoemulsification and hydrophilic acrylic IOL (Akreos MI60) implantation in his right eye 18 months ago. At 1 month after the cataract surgery, his UCVA was 20/63 and his BCVA was 20/20 with an SE of − 2.00 D. At the time of presentation, his UCVA and BCVA were 20/250 and 20/100, respectively, and his SE was + 0.375 D. The IOP and anterior ocular surface did not show any abnormality. Slit lamp examination after dilation revealed marked shrinking of the anterior capsular opening. An Nd:YAG laser anterior capsulotomy was performed. One month after the Nd:YAG laser treatment, his UCVA and BCVA improved to 20/100 and 20/20, respectively, and his SE value returned to – 1.375, which was similar to the value after the cataract surgery. Twelve months later, his BCVA was 20/20 in the right eye, without recurrence.

### Case 3

A 66-year-old woman with no systemic disease visited our clinic due to decreased visual acuity in her right eye. She underwent phacoemulsification surgery with IOL (Akreos MI60) implantation 2 months ago. Her postoperative UCVA and BCVA were 20/100 and 20/25, respectively, and her SE was − 2.00 D. At the time of presentation, her UCVA and BCVA were 20/100 and 20/50 respectively. On a refraction test, her SE was − 0.125 D, which showed a hyperoptic shift compared to the value immediately after her cataract surgery. Dilated slit lamp examination revealed phimosis of the anterior capsule with posterior vaulting of the IOL optic. Laser anterior capsulotomy was performed with an Nd:YAG laser in the right eye. One month after the Nd:YAG treatment, her UCVA and BCVA improved to 20/63 and 20/32, respectively. On a refraction test, the SE was − 1.50 D. Eight months later, her UCVA and BCVA were 20/63 and 20/32, respectively, in the right eye, without any complications.

### Case 4

A 76-year-old woman who had phacoemulsification cataract extraction 6 months ago was referred to our clinic due to decreased visual acuity in the right eye. She had controlled hypertension and diabetes. At the time of the cataract surgery, a Akreos MI-60 IOL was implanted in the capsular bag. Postoperative UCVA and BCVA were 20/32 and 20/20, respectively. The postoperative refraction test showed that her SE was + 0.50 D. At the time of presentation, UCVA and BCVA were 20/50 and 20/25, respectively, and the SE showed a hyperoptic shift of + 1.875 D. Dilated slit lamp examination revealed 360 degrees of anterior capsular phimosis. Nd:YAG laser anterior capsulotomy was used to create a radial opening in the capsular phimosis. One month after the Nd:YAG treatment, her UCVA and BCVA improved to 20/25 and 20/20. The refraction test showed an SE of + 0.75 D. Twelve months later, her UCVA and BCVA were 20/20 in the right eye. There was no sign of anterior capsular contraction in the right eye.

## Discussion

CCS can affect vision not only because of opacification of the visual axis but also because of IOL decentration such as tilting, deformation of the IOL, hypotony, and retinal detachment [1, 18, 21]. Increasing interest in microincision cataract surgery has led to the use of more flexible IOLs that can be inserted through smaller corneal incisions. However, the more flexible the IOL is, the more physical deformation and refractive error can occur when CCS occurs. In this study, we have reported the case of 4 patients with a hyperopic shift caused by CCS after phacoemulsification followed by hydrophilic acrylic Akreos MI60 IOLs implantation in the capsular bag.

CCS is caused by lens epithelial cell proliferation. Spang et al. [22] reported that excessive capsule shrinkage is most likely caused by actin filaments in residual lens epithelial cells. In the presence of weak zonular support, actin filament contraction can result in CCS development. Remnant lens epithelial cells undergo a myofibroblastic transformation, with altered cells containing smooth muscle actin, and contraction occurs in the resultant fibrous membrane. Such anterior capsule contraction occurs more often in the presence of zonulysis, which causes centripetal forces to be larger than centrifugal forces.

CCS can be provoked by a number of contributing factors such as capsulorhexis size, high myopia, retinitis pigmentosa, uveitis, diabetes, and IOL material and design.1–4 In this study, capsulorhexis size did not likely have a large impact on CCS incidence because the 4 patients included in our study had a similar capsulorhexis size (range: 5.5–6.0 mm). Additionally, all procedures were performed by a single surgeon. Therefore, it is presumed that the surgical factors did not have a significant influence on the occurrence of CCS in our study. The CCS patients did not have high myopia or ocular inflammatory disease. Two patients had diabetes, which may have been a risk factor, but CCS did not occur earlier than in the patients without diabetes. There were no differences in the axial length, anterior chamber depth, or preoperative and postoperative SE between the affected eyes in the 4 patients with CCS and the 295 eyes without CCS (Table 1). Therefore, we conclude that ocular factors did not seem to influence CCS occurrence. However, it should be noted that this study only included 4 cases and further verification is needed.

All patients in this case series underwent in-the-bag implantation of hydrophilic Akreos MI60 IOL. It has been reported that the adhesion and proliferation of lens epithelial cells is more active with the use of hydrophilic acrylic IOLs than with the use of hydrophobic acrylic material, and the incidence of CCS is higher in case of hydrophilic acrylic IOLs implantation [23–25]. Tsinopoulos et al. [25] reported that CCS was significantly greater after hydrophilic IOL implantation than after hydrophobic IOL implantation. However, Richter et al. [26] reported a similar CCS incidence with hydrophilic acrylic, hydrophobic acrylic, and silicone IOLs. Cochener et al. [27] reported that CCS occurs more frequently with silicone IOLs than with polymethylmethacrylate (PMMA) IOLs. Mingels et al. [28] reported that CCS occurs more frequently following implantation of IOLs with fewer haptics following implantation of one piece, hydrophilic acrylic IOLs with a different number of haptics. However, Tsinopoulos et al. [26] did not find a significant correlation between the number of haptics and CCS incidence. The IOL design may also impact CCS incidence. Sacu et al. [29] reported that CCS incidence increased as the optical margin thickness decreased. Thus, various results have been reported as to whether the IOL material and design influence the occurrence of CCS and it is difficult to conclude. We do not speculate that the materials of the Akreos IOL has affected the occurrence of CCS in our study. Because many previous studies have shown that CCS can occur in IOLs of various materials [5, 8–20]. However, in the case microincision cataract surgery with IOLs made of flexible materials, like the Akreos MI60 IOL, the occurrence of IOL deformation and decentration may increase when CCS occurr. This also causes refractive changes and affects the quality of vision.

**Fig. 2:**
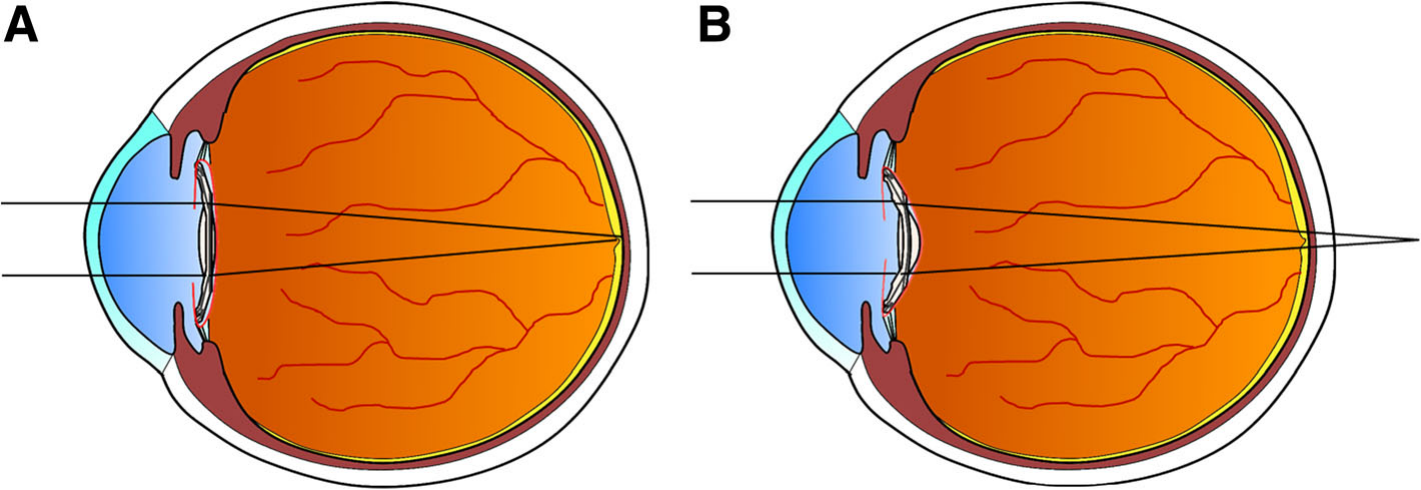
Illustration of posterior bowing of intraocular lens after capsule contraction syndrome. **a** Before capsule contraction syndrome occurs, parallel light is focused on the fovea. **b** After capsule contraction syndrome, hyperopic shift occurs due to posterior bowing of the intraocular lens. The diagram was illustrated by Dr. Tae Gi Kim using Paint.NET software (Version 4.1)

In this study, IOL deformation occurred as a contraction of the capsular bag when CCS occurred, which caused an average hyperopic shift of 1.78 ± 0.45 D compared with that after cataract surgery. A possible mechanism for this hyperopic shift in refraction is that as the capsular bag contracts, the IOL is pressurized in a limited space forcing it to eventually bows backwards (Fig. 2). Asymmetrical vaulting caused by CCS can result in IOL tilting, which causes astigmatism. Two of the 4 cases included in this study had an increase in astigmatism after CCS development. However, the amount of change was less than 1.0 D. Therefore, hyperopic shifts caused by CCS more likely resulted from IOL posterior vaulting than from an astigmatism increase. Ozturk et al. [12] reported that because the Collamer CC420BF (Staar Surgical) IOL is highly flexible, the mild equatorial contraction may have resulted in posterior vaulting. Sanders et al. [10] reported that the most likely etiology of hyperopic shift after implantation of the collamer plate-haptic IOL was the development of anterior capsule fibrosis. Qatarneh et al. [17] suggested the posterior movement of the IOL due to CCS as a mechanism of hyperopic shift that occurred between 6 months and 1 year after implantation of the Akreos Adapt IOL (Bausch & Lomb). Therefore, when using flexible material IOLs in microincision cataract surgery, refraction changes should be carefully monitored.

Various treatments options for CCS include Nd:YAG laser anterior capsulotomy, surgical enlargement of the phimosis (using a vitreous cutter or capsule scissors), and forceps enlargement of the phimosis with IOL scleral fixation [5, 8–20]. If capsule contraction is severe and the zonules are weak, IOL exchange with IOL iris fixation or IOL insertion into the anterior chamber may be performed [12, 17]. Capsulotomy enlargement with a femtosecond laser was also recently performed [30], but Nd:YAG lasers are more commonly used because the surgery is less challenging.

To our knowledge, there are few studies on refractive changes after CCS treatment. Qatarneh et al. [17] reported that Nd:YAG laser anterior capsulotomy did not restore the position of the IOL and proper refraction. In the series described by Sanders et al., 50% cases with capsule phimosis treated with Nd:YAG laser anterior capsulotomies had a reduction in hyperopic shift [10]. In our study, hyperopic shift was reduced in all cases after Nd:YAG laser anterior capsulotomy. Through these results, we confirmed that Nd:YAG laser anterior capsulotomy is an effective treatment option for improving visual acuity and reducing refractive error in patients with CCS.

This study was limited by its small sample size. Therefore, our study findings cannot be generalized. Another limitation is that the anterior chamber depth of the patient with CCS was estimated by an anterior photograph or a medical record of slit lamp examination. Different angles of the incident slit beam can affect anterior chamber depth estimation. Further studies that include a larger number of patients and measure anterior chamber depth through anterior segment optical coherence tomography or ultrasound biomicroscopy are needed to confirm our results. However, it is meaningful that eyes with CCS and flexible IOLs tended to be vulnerable to hyperopic refractive shift.

## Conclusion

In conclusion, when microincision cataract surgery was performed with with Akreos MI60 IOL implantation, IOL deformation due to capsular bag contraction during CCS may cause hyperopic shift. Nd:YAG laser anterior capsulotomy can effectively restore vision and correct the refractive error in cases with CCS.

## Abbreviations

BCVA: Best corrected visual acuity
CCS: Capsule contraction syndrome
IOL: Intraocular lens
SE: Spherical equivalent
UCVA: Uncorrected visual acuity
